# Biomechanics over body composition: what matters most in the 40

**DOI:** 10.1080/15502783.2025.2550184

**Published:** 2025-08-25

**Authors:** Emma Sterne, Chukwudum Okpalaoka, Pavan Patel, Alexa Fraga, Caitlin Kucharski, Pete Bommarito, Monique Mokha

**Affiliations:** a Nova Southeastern University, Lauderdale, FL, USA; b Bommarito Performance Systems, Davie, FL, USA

**Keywords:** Energy availability, menstruation, nutrition, metabolic rate

## Abstract

**Background:**

The 40-yard dash (“the 40”) is the most anticipated event at the National Football League’s (NFL) annual combine. Lower times are associated with higher draft position, and drafted players outperform non-drafted players. Biomechanical markers of linear speed include short ground contact times (GCTs) and large vertical ground reaction forces (vGRFs) while force production during sprinting is influenced by improvements in lean muscle mass (LMM) and body fat (BF%). Thus, coaches and sports scientists spend considerable resources addressing these factors, but often separately. Additionally, many draft eligible athletes have relatively little time to transition from gameplay to testing. Thus, this study aimed to develop a multiple linear regression model to estimate 40-yard run times of NFL draft eligible players undergoing a structured 6-week training program using running biomechanics (GCT, vGRF) and body composition (LMM, BF%) dependent variables.

**Methods:**

This study was part of a larger investigation into performance monitoring of 75 American football players participating in a 6-week NFL draft preparation training camp. Data from 39 were selected. All participants had just completed their collegiate playing season, were active players training 5–6 times per week, and had a scheduled NFL try-out. LMM and BF% were measured using an InBody 270 in accordance with the manufacturer’s specifications. After a 25-min standardized warm-up, running mechanics were captured in a laboratory using an instrumented treadmill synchronized with a 3-D motion analysis system. Treadmill speed increased 1 m/s in 1-s increments until the preferred maximum speed was reached (~6.5 m/s),g at which the participants ran for 1- to 2-s before a 5-s recording was taken. 5-s was selected to encompass most 40 times for this population. The running biomechanics data yielded were GCT and peak vGRF. The tests were repeated at the end of camp. The 40 times were measured with laser timers and obtained from publicly available data from the NFL and the director of the training camp, who monitored pro day performances. A multiple linear regression model was developed using the stepwise technique to predict post-training 40 times from post minus pre changes (Δ) in running biomechanics and body composition variables, *p* < .05.

**Results:**

The fitted regression model was 4.654 – 0.972 * (ΔLMM) − 0.961 * (ΔBF%) − 10.078 (ΔGCT) + 0.048 * (ΔvGRF). The model significantly predicted 40-yard dash performance (*R* = .655, R^2^ = .429, F(4,34) = 6.397, *p* < .001). However, among the predictors, only changes in GCT were significantly associated with improvements in 40 time (β = −10.08, *p* < .001). Changes in BF%, LMM, or vGRF did not reach statistical significance.

**Conclusion:**

These findings suggest that the reductions in GCT, rather than changes in body composition or force applied during running, are more strongly associated with improved sprint performance in NFL draft prospects. While body composition should not be neglected, training programs may benefit from emphasizing neuromuscular and biomechanical strategies to reduce GCT.

